# Cortical gyrification and its relationships with molecular measures and cognition in children with the *FMR1* premutation

**DOI:** 10.1038/s41598-020-73040-0

**Published:** 2020-09-29

**Authors:** Jun Yi Wang, Merna Danial, Cyrus Soleymanzadeh, Bella Kim, Yiming Xia, Kyoungmi Kim, Flora Tassone, Randi J. Hagerman, Susan M. Rivera

**Affiliations:** 1 Center for Mind and Brain, University of California-Davis, 267 Cousteau Place, Davis, CA 95618 USA; 2 MIND Institute, University of California-Davis Medical Center, Sacramento, CA 95817 USA; 3 Department of Biochemistry and Molecular Medicine, School of Medicine, University of California-Davis, Sacramento, CA 95817 USA; 4 Department of Psychology, University of California-Davis, Davis, CA 95616 USA; 5 Department of Public Health Sciences, School of Medicine, University of California-Davis, Sacramento, CA 95817 USA; 6 Department of Pediatrics, School of Medicine, University of California-Davis, Sacramento, CA 95817 USA

**Keywords:** Brain, Diagnostic markers, ADHD, Anxiety, Paediatric research

## Abstract

Neurobiological basis for cognitive development and psychiatric conditions remains unexplored in children with the *FMR1* premutation (PM). Knock-in mouse models of PM revealed defects in embryonic cortical development that may affect cortical folding. Cortical-folding complexity quantified using local gyrification index (LGI) was examined in 61 children (age 8–12 years, 19/14 male/female PM carriers, 15/13 male/female controls). Whole-brain vertex-wise analysis of LGI was performed for group comparisons and correlations with IQ. Individuals with aberrant gyrification in 68 cortical areas were identified using *Z*-scores of LGI (hyper: Z ≥ 2.58, hypo: Z ≤ − 2.58). Significant group-by-sex-by-age interaction in LGI was detected in right inferior temporal and fusiform cortices, which correlated negatively with CGG repeat length in the PM carriers. Sixteen PM boys (hyper/hypo: 7/9) and 10 PM girls (hyper/hypo: 2/5, 3 both) displayed aberrant LGI in 1–17 regions/person while 2 control boys (hyper/hypo: 0/2) and 2 control girls (hyper/hypo: 1/1) met the same criteria in only 1 region/person. LGI in the precuneus and cingulate cortices correlated positively with IQ scores in PM and control boys while negatively in PM girls and no significant correlation in control girls. These findings reveal aberrant gyrification, which may underlie cognitive performance in children with the PM.

## Introduction

The fragile X premutation (PM) is characterized by a 55–200 CGG repeat expansion in the 5′ untranslated region of the fragile X mental retardation 1 (*FMR1*) gene located on the X-chromosome^1^. The mutation is associated with a spectrum of clinical conditions affecting PM carriers across the lifespan. Two associated disorders have been identified, namely: fragile X-associated tremor/ataxia syndrome (FXTAS) that occurs in 45% of male PM carriers and 16% of female PM carriers after age 50^2,3^ and fragile X-associated primary ovarian insufficiency (FXPOI) that affects 16–20% female PM carriers before age 40. Fragile X-associated neuropsychiatric disorders (FXAND) is a newly proposed group of disorders that may affect a subset of children and adults with the PM^4^. Although the prevalence of developmental delay is not increased in the PM population^5^, a national parent survey of 1276 families of children with the fragile X syndrome indicates that children with the PM have increased risks to be diagnosed or treated for anxiety, attention-deficits/hyperactivity disorder (ADHD), and social impairment compared with age and family-income matched controls^6^. Both probands who are clinically referred and nonprobands who are identified as a result of family investigation have been shown to present a higher rate of involvement compared with non-carrier controls or the general population^6–9^. In adult PM carriers, anxiety, depression, ADHD, obsessive compulsive disorder (OCD), and substance abuse are the most common types of neuropsychiatric conditions^8–14^. Sensory difficulties can occur as early as in infants with the PM, who displayed hypersensitivities that have a potential to be clinically relevant later in life^15,16^. Cognitively, a higher threshold level in motion contrast detection compared with typically developing infants has been reported^15–17^. Additionally, adult PM carriers without FXTAS showed longer manual movement and reaction times^18^ as well as subtle impairment in executive function and declarative learning and memory performance^19,20^. In a non-clinical referred sample of 64 young PM carriers who were unaffected by FXTAS and without children with fragile X full mutation, higher rates of autism-related symptoms in males and high rates of obsessive–compulsive symptoms in female compared with age- and sex-matched non-carrier controls have been reported^10^. FMRP deficiency may also be associated with higher rates of anxiety disorders since the percentage is higher in carriers with intellectual disability (ID) (81.8%) compared to carriers with normal IQ (62.5%)^8^. Significant correlations between elevated *FMR1* mRNA level and obsessive–compulsive symptoms and psychoticism have also been reported in adult PM carriers, supporting the role of the RNA-toxicity in neuropsychiatric conditions. Other potential mechanisms for neuropsychiatric vulnerabilities include rare copy number variation detected in PM children with behavior and/or neuropsychiatric problems^21^, mitochondrial dysfunction^22,23^, chronic DNA damage repair^24^, and the presence of FMRPolyG, the toxic protein product of the repeat-associated non-AUG (RAN) translation of the expanded-CGG containing mRNA^25^.

Despite the accumulating evidence of the elevated risk of developmental problems, neurobiological substrates of cognitive development and neuropsychiatric conditions have not been investigated in children with the premutation. The knock-in mouse models of the PM have provided clues suggesting that altered neocortical development may occur as early as during late embryogenesis. A study of embryonic neocortical development in PM mice^26^ detected FMRP expressions in both radial glial cells in the ventricular zone and neural precursor cells in the subventricular zone albeit the overall FMRP expression in the neocortex was slightly reduced compared with the wild-type. The lower FMRP expression may lead to delayed differentiation of neocortex neurons and impaired neuronal migration observed in the embryonic PM neocortex^26^. In addition, the level of expanded CGG-containing *FMR1* mRNA is elevated in PM carriers (i.e., the RNA toxicity), which may sequester proteins important for neurodevelopment^27–29^. Demonstrated in a recent study of PM mice, reducing *FMR1* mRNA levels using small interfering RNA (siRNA) normalized the expression of 29 proteins including RNA-binding proteins and mitochondrial proteins, and rescued alterations in dendritic morphology in cultured hippocampal neurons^30^. These findings support the pathogenic role of the RNA toxicity affecting brain structure and function. Although it cannot be directly observed in lissencephalic species such as mice, cortical folding or gyrification may be affected in PM carriers. Formed mainly during the 4th fetal month to first postnatal year in humans, gyrification is a complex process under the control of tightly regulated gene expression in both temporal and spatial dimensions^31^. Perturbations of gene expression during this period are found to affect neuronal proliferation, migration, and differentiation, which would ultimately result in altered gyrification^32^.

To understand neurobiological bases for cognitive development and neuropsychiatric conditions in PM carriers, we examined cortical folding in children with the PM and its relationships with *FMR1* molecular measurements and cognitive functioning. Given the variability found in the phenotype of children with the PM^4^, we explored gyrification analyses at both group and individual levels to account for potential spatial and directional (i.e., hyper- or hypo-gyrification) variations in gyrification. Gyrification was quantified using local gyrification index (LGI)^33^, the ratio of total surface area (both hidden within the sulci and exposed on the outer surface of the brain) and exposed surface area. The comparison measurements were cortical thickness (CT) and surface area (SA), which are likely to also be affected by the PM.

## Results

We examined brain scans from 61 children–33 PM carriers (19 boys, 14 girls) and 28 age-matched controls (15 boys, 13 girls) aged 8–12 years (Table 1). Visual inspection of the MRI scans did not detect any scans with excessive motion artifacts that would affect LGI-quantifications. To further assure image quality, we obtained Euler number generated by the Freesurfer software package^34^, which summarizes topological complexity of the cerebral cortex but has been shown to correlate with manual identification of “unusable” scans^35^, as well as nine LONI Quality Control (QC) metrics that were unlikely to be affected by topological complexity^36^. The nine LONI metrics comprised (1) mean slice intensity (MSI), (2) signal to noise ratio (SNR), (3) signal variance-to-noise variance ratio (SVNR), (4) contrast-to-noise ratio (CNR), (5) contrast of variance-to-noise ratio (CVNR), (6) brain tissue contrast-to-tissue intensity variation (TCTV), (7) full-width-at-half-maximum (FWHM), (8) center of mass (CoM), and (9) coefficient of variation (COV). To determine the cutoffs for “unusable” scans, we utilized two scans acquired from a 7-year-old male control and a 13-year-old PM male, who were outside of the age range and showed excessive motion artifacts. The Euler numbers were − 330 and − 468, respectively (Supplementary Fig. S1a,b). In the current dataset, only the Euler number of − 334 (Supplementary Fig. S1c) overlapped with those of the two “unusable” scans. Of the nine QC metrics from LONI, only SNR and COV were able to separate the two “unusable” scans from the remaining scans with no overlapping (SNR: *z* = − 3.2 and − 2.5, COV: *z* = − 2.8 and − 2.5) while the other seven metrics were nowhere near useful (i.e.,* z*-scores close to mean or in the wrong direction). However, the scan with the Euler number of − 334 had *z*-scores of SNR and COV at − 0.43 and − 0.48, respectively. To avoid excluding scans because of topological complexity we decided to include this scan. To take into account of the potential effect of movement on the results, we reported results with and without using the Euler number as a covariate for analyzing CT and SA.

**Table 1:** Characteristics of research participants: mean (SD).

	*N*	Male controls	*N*	Male PM carriers	Test statistics	*N*	Female controls	*N*	Female PM carriers	Test statistics
Age	15	10.6 (1.9)	19	10.6 (1.3)	*t* = − 0.04, *p* = 0.97	13	9.9 (1.6)	14	10.7 (1.7)	*t* = 1.25, *p* = 0.22
CGG repeat size	14	30.0 (4.2)	19	100 (37.5)	*t* = 8.09, *p* < 0.001	11	26.5 (4.2) 30.3 (2.5)	14	30.7 (7.7) 95.6 (30.8)	*t* = 1.76, *p* = 0.09 *t* = 7.90, *p* < 0.001
*FMR1* mRNA	14	1.31 (0.17)	19	2.77 (0.92)	*t* = 6.71, *p* < 0.001	8	1.44 (0.30)	13	2.45 (1.00)	*t* = 3.42, *p* = 0.004
WISC-IV FSIQ	13	115.3 (11.3)	18	92.3 (17.0)	*t* = − 4.53, *p* < 0.001	11	110.6 (8.7)	14	110.4 (15.6)	*t* = − 0.04, *p* = 0.97
WISC-IV VCI	13	119.8 (11.2)	18	95.8 (17.2)	*t* = − 4.70, *p* < 0.001	11	112.5 (14.1)	14	113.0 (14.4)	*t* = − 0.08, *p* = 0.94
WISC-IV PRI	13	113.8 (12.2)	18	98.3 (13.6)	*t* = − 3.35, *p* = 0.002	11	111.4 (11.6)	14	113.4 (12.0)	*t* = 0.42, *p* = 0.68
WISC-IV WMI	13	103.6 (12.7)	18	89.3 (16.1)	*t* = − 2.77, *p* = 0.010	10	103.2 (9.6)	14	101.9 (17.2)	*t* = − 0.24, *p* = 0.81
WISC-IV PSI	13	103.6 (9.9)	18	87.9 (13.0)	*t* = − 3.82, *p* < 0.001	10	100.8 (12.7)	14	98.9 (15.8)	*t* = − 0.33, *p* = 0.74

Among the 33 PM carriers, nine boys and two girls were probands while the remaining PM carriers were nonprobands (Supplementary Table S1). WISC-IV scores were obtained from 56 participants, which were significantly lower in male PM carriers than male controls (*p* < 0.001–0.010) but showed no differences between the female PM carriers and controls (*p* > 0.05) (Table 1). The medical history revealed 80.0% PM carriers (17/19 boys and 7/11 girls) who were diagnosed with anxiety disorder, and 50% (12/19 boys and 3/11 girls) who showed ADHD symptoms. All PM carriers with ADHD were comorbid with anxiety disorder except for one boy. However, the rate of anxiety, ADHD, and FSIQ < 80 were not preferentially higher in the probands compared with the nonprobands (Supplementary Table S2).

### Group-by-sex-by-age interaction in LGI and CT

**Figure 1:**
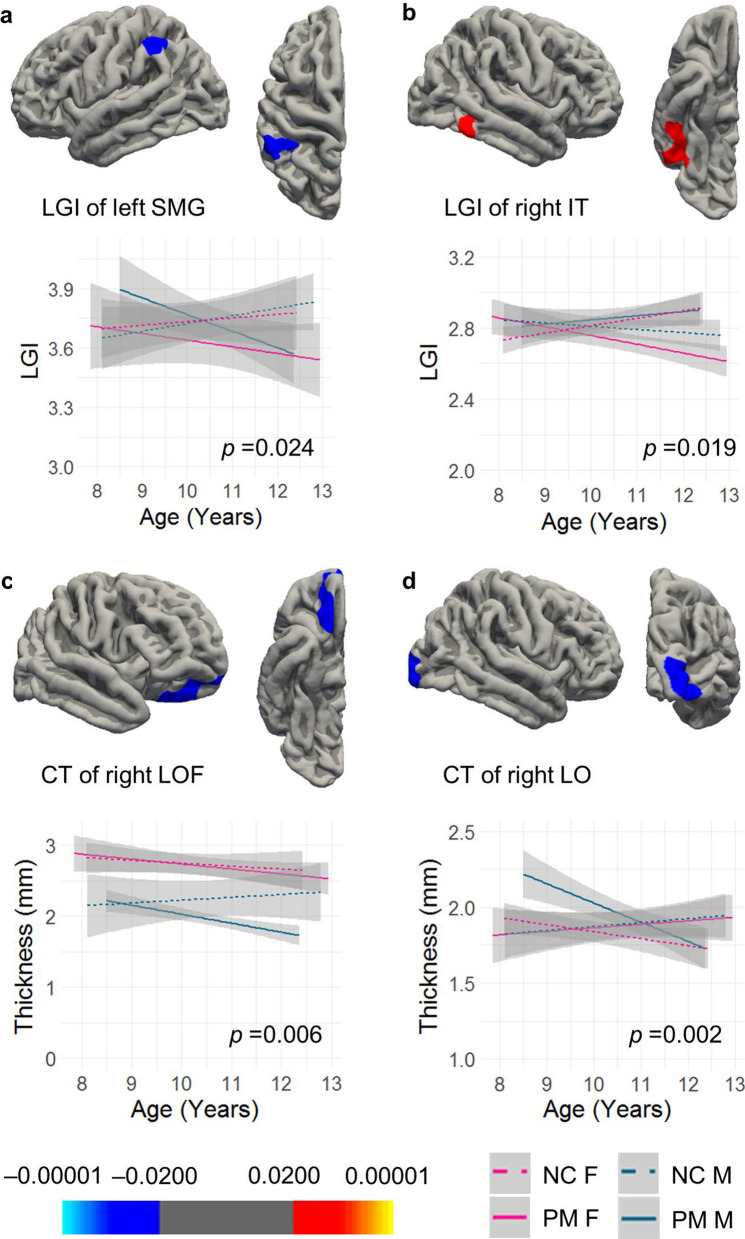
Vertex-wise group comparisons of LGI and CT. (**a**) A cluster peaked at the left supramarginal gyrus (SMG) showing group-by-age intereaction in LGI. (**b**) A cluster peaked at the right inferior temporal cortex (IT) showing group-by-sex-by-age interaction in LGI. (**c**) A cluster peaked at the right lateral orbitofrontal cortex (LOF) showing group effect in CT. (**d**) A cluster peaked at the right lateral occipital cortex (LO) showing group-by-sex-by-age interaction in CT. All clusters were formed using the CFT of *p* = 0.010. Cluster-wise *p* values are shown in the graph.

In whole brain vertex-wise analysis of LGI, three levels of cluster-forming threshold (CFT) (0.05, 0.01, and 0.005) were tested and reported. Using CFT of *p* = 0.010 revealed one cluster in the supramarginal cortex extending to the inferior parietal cortex (LGI1) displaying significant group-by-age interaction (*p* = 0.024) and another cluster in the right inferior temporal cortex extending to the fusiform (LGI2) showing significant group-by-sex-by-age interaction (*p* = 0.019) (Table 2, Fig. 1a,b). The analysis of the comparison variable, CT, detected a cluster in the right lateral orbitofrontal cortex extending to the frontal pole and medial orbitofrontal cortices (CT1) showing significant group effect (*p* = 0.006) (Fig. 1c). In addition, a cluster in the right lateral occipital cortex (CT2) showed significant group-by-sex-by-age interaction (*p* = 0.002) (Table 2, Fig. 1d). Two of the clusters, LGI2 and CT2, also survived the more stringent CFT threshold of *p* = 0.005 but with a smaller cluster size compared with CFT of *p* = 0.010 (Table 2). In contrast, no clusters showed any significant effect in SA. Using CFT of *p* = 0.050, two additional LGI clusters showed group-by-sex-by-age and group-by-age interactions, respectively, and one cluster of SA showed a group-by-age interaction (Table 2).

**Table 2:** Cortical regions showing significant effect in group analysis using 10 mm smooth kernals for CT and SA.

Finding	Brain region	Size (mm^2^)	Talairach coordinates (x, y, z)	Cluster-wise *P*
**Clusters survived CFT of p = 0.010 (Euler number not used as a covariate)**				
LGI1: group-by-age	Left supramarginal cortex, extending to inferior parietal cortex	970.67	− 56.9, − 40.3, 38.2	0.0239
LGI2: group-by-sex-by-age	Right inferior temporal cortex, extending to fusiform	970.01	49.7, − 47.2, − 15.8	0.0190
CT1: group	Right lateral orbitofrontal cortex, extending to frontal pole and medial orbitofrontal cortex	558.99	14.7, 24.9, − 24.7	0.0056
CT2: group-by-sex-by-age	Right lateral occipital cortex	628.29	23.4, − 98.1, 1.9	0.0020
**Clusters survived CFT of p = 0.005**				
LGI2: group-by-sex-by-age	Right inferior temporal cortex, extending to fusiform	793.15	49.7, − 47.2, − 15.8	0.0116
CT2: group-by-sex-by-age	Right lateral occipital cortex	503.96	23.4, − 98.1, 1.9	0.0012
**Additional clusters passed CFT of p = 0.050 (Euler number not used as covariate)**				
LGI: group-by-sex-by-age	Left inferior temporal cortex, extending to fusiform, lateral occipital, lingual, and middle temporal cortices	4448.51	− 46.2, − 61.9, − 6.2	0.0002
LGI: group-by-age	Right inferior parietal cortex, extending to supramarginal, superior parietal, and postcentral cortices	2013.77	44.1, − 60.6, 45.5	0.0241
SA: group-by-age	Right rostral middle frontal cortex, extending to superior frontal and frontal pole cortices	2587.86	28.3, 41.4, 18.2	0.0016
**Clusters passed CFT of p = 0.050 (Euler number used as covariate)**				
CT: group	Right lateral orbitofrontal cortex, extending to pars orbitals, pars triangularis, and insula cortices	1177.73	16.2, 27.3, − 23.8	0.0056
CT: group-by-age	Right superior parietal cortex, extending to inferior parietal, and precuneus cortices	1109.23	21.8, − 62.7, 50.6	0.0064
**Euler effect: clusters survived CFT of p = 0.010**				
LGI	Left precentral cortex, extending to caudal middle frontal, postcentral, superior parietal, supramarginal cortices	3899.52	− 43.6, − 7.2, 45.8	0.0002
LGI	Right inferior parietal cortex, extending to supramarginal, superior parietal, and superior temporal cortices	3146.97	45.7, − 49, 11.5	0.0002
LGI	Right rostral middle frontal cortex, extending to pars opercularis and pars triangularis	858.76	43.7, 28.5, 22.4	0.0056
SA	Right superior frontal cortex, extending to rostral middle frontal cortex	1896.9	− 7.6, 60.1, 20.4	0.0002

We also tested the effect of Euler on cortical measures and repeated the CT and SA analyses after adding the Euler number as a covariate. Three LGI clusters survived the CFT of 0.01, showing negative correlation with the Euler number centered in left precentral cortex, right inferior parietal cortex, and right rostral middle frontal cortex (Table 2, Supplementary Fig. S2a,b). In contrast to no Euler effect on CT even at CFT of 0.05, an SA cluster passed CFT of 0.01 centered at left superior frontal cortex (Table 2, Supplementary Fig. S2c). All of the three LGI clusters and the SA cluster also survived the more stringent CFT of 0.005. After controlling for the Euler number, only two CT clusters passed CFT of 0.05, showing group effect centered at right lateral orbitofrontal cortex and group-b-age interaction centered at right superior parietal cortex (Table 2). No significant clusters of SA were found at any CFT level. The above analyses were repeated after excluding the 4 scans acquired using a 32-channel head coil from one male control, one female PM carrier, and two male PM carriers. The same clusters with similar sizes and levels of significance were detected.

Post-hoc analyses were performed to study the associations between the average LGI of the significant clusters and CGG repeat length and *FMR1* mRNA level, using multiple linear regression. Age and total cranial volume (TCV) were included as covariates. For female PM carriers, CGG repeat size of the premutation allele and the activation ratio (AR), indicating the percentage of cells carrying the normal allele on the active X chromosome, were included in the analysis. The analyses revealed a significant negative association between cluster LGI2 with CGG repeat length in male PM carriers (*β* = − 0.0014 ± 0.0005, *p* = 0.010) (Supplementary Fig. S3a). Significant correlations between LGI2 and CGG repeat length (*β* = − 0.0019 ± 0.0004, *p* = 0.001) and the activation ratio (*β* = − 0.29 ± 0.064, *p* = 0.002) were also demonstrated in female PM carriers after excluding a girl showing an outlier effect (Supplementary Fig. S3b,c). LGI of this cluster exhibited a negative correlation with CT (*r* = − 0.27, *p* = 0.038) and a positive correlation with SA (*r* = 0.32, *p* = 0.013) combining all four groups without the adjustment for age and TCV.

### Majority (78.8%) of the PM carriers showed aberrant gyrification

**Figure 2:**
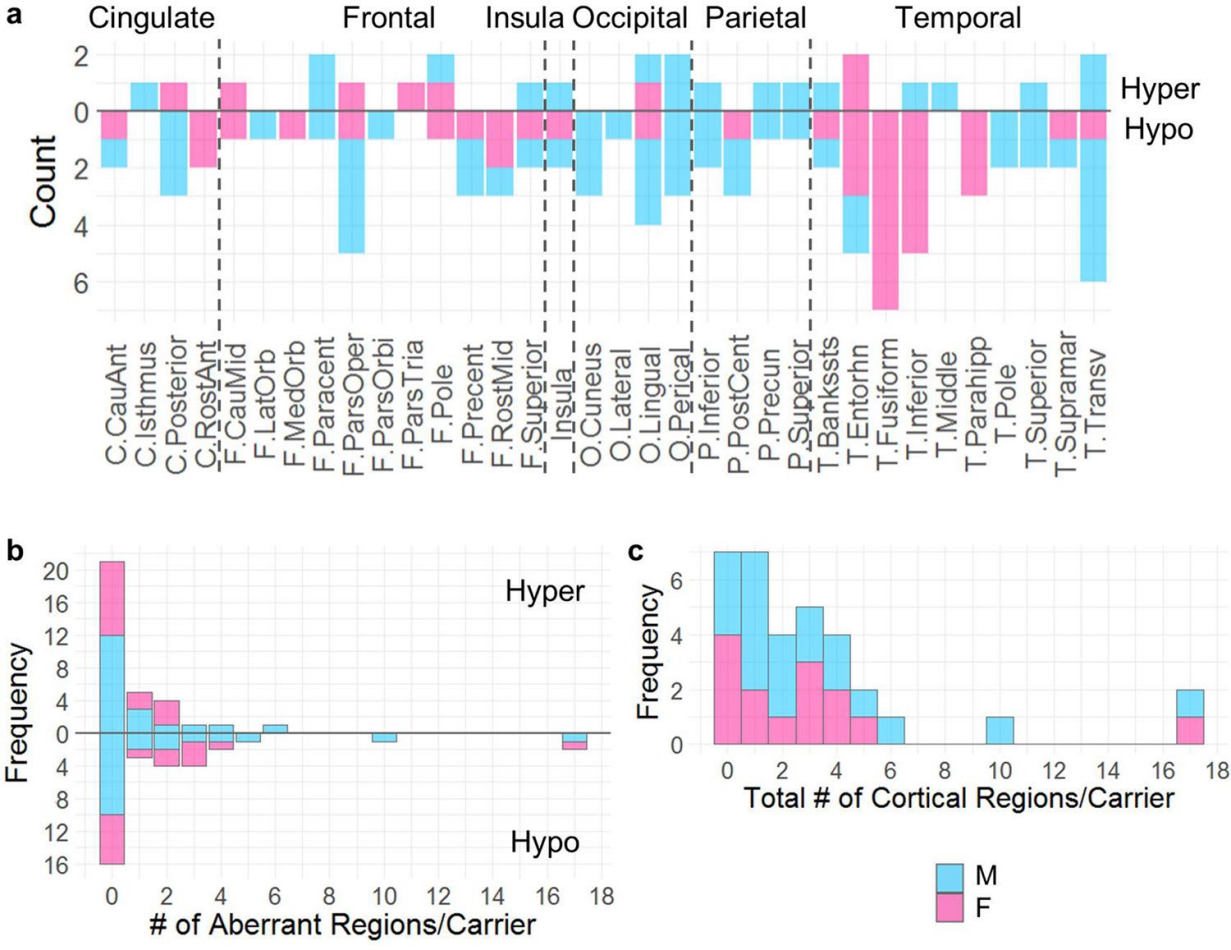
Hyper- and hypo-gyrification in individual PM carriers. (**a**) Number of male and female PM carriers showing hyper- and hypo-gyrification in the 34 cortical regions (left and right are combined). C, cingulate cortex; F, frontal lobe; O, occipital lobe; P, parietal lobe; T, temporal lobe. (**b**) Frequency of PM carriers with cortical regions showing hyper- versus hypo-gyrification. (**c**) Frequency of PM carriers with cortical regions showing either hyper- or hypo-gyrification. M, male PM carrier; F, female PM carrier.

Individuals with Z-scores above 2.58 and below − 2.58 were considered as having hyper- and hypo-gyrification, respectively. The analysis of average LGI of 68 cortical regions demonstrated that 78.8% PM carriers (16/19 boys, 10/14 girls) showed 1–17 regions with hyper- or hypo-gyrification in contrast to 14.3% controls (2/15 boys, 2/13 girls) showing one such region per person. Regions with aberrant gyrification affected all lobes in male PM carriers but occurred in higher frequencies in the temporal regions as compared with other cortical regions in female PM carriers (Fig. 2a). Seven male PM carriers and 5 female PM carriers showed hypergyrification in 1–6 regions (36.4%, *Z* = 2.58–5.33) while only one female control showed hypergyrification, and only in one region (3.6%, *Z* = 2.94). In addition, 9 male PM carriers and 8 female PM carriers showed hypogyrification in 1–17 regions (51.5%, *Z* = − 5.39 to − 2.58) while only two male controls and one female control showed hypogyrification, and in only one region per person (10.7%, *Z* = − 2.71 to − 2.59). Three of the 8 female PM carriers showed both hypogyrification (2–3 regions/person) and hypergyrification (1–2 regions/person). Figure 2b and c depicts the frequency of PM carriers with a specific number of aberrant regions. The group comparisons showed significantly higher percentages of hypergyrification regions, hypogyrification regions, and total aberrant gyrification regions in PM carriers than those of the controls (*p* < 0.0001–0.0008) (Table 3).

**Table 3:** Comparisons of number of cortical regions with aberrant gyrification between carriers and controls.

	Controls (*N* = 28)		Carriers (*N* = 33)		Test statistics
	Mean (SD)	Range	Mean (SD)	Range	
Hypergyrification (%)	0.1 (0.3)	0–1.5	1.2 (2.0)	0–8.8	*t* = − 3.62, df = 40.6, *p* = 0.0008
Hypogyrification (%)	0.2 (0.5)	0–1.5	3.6 (6.3)	0–25.0	*t* = − 4.19, df = 48.4, *p* = 0.0001
Total aberrant (%)	0.2 (0.5)	0–1.5	4.7 (6.1)	0–25.0	*t* = − 7.12, df = 56.7, *p* < 0.0001

The *t*-tests were performed on log transformed data after adding 0.0001 to accommodate zero percentage of regions with aberrant LGI in some participants.

We also observed that 9 male PM carriers and 2 female PM carriers had regions of aberrant LGI containing the primary sensorimotor cortices (i.e., precentral, postcentral, transverse temporal, and pericalcarine cortices) (primary group) while the remaining 7 male and 8 female PM carriers showed aberrant LGI only in the association cortices (association group). A comparison between the two groups revealed higher proportions of aberrant LGI regions in the primary group than the association group for PM boys [primary/associative: mean(SD) = 0.088(0.070)/0.019(0.007), range 0.029–0.25/0.015–0.029, *t* = 2.96, df = 8.20, *p* = 0.009] while the comparison did not show a significant difference in PM girls [primary/associative: mean(SD) = 0.147(0.022)/0.042(0.022), range 0.044–0.25/0.015–0.074, *t* = 1.013, df = 1.011, *p* = 0.247].

### Cortical morphology correlated with cognitive performance

**Figure 3:**
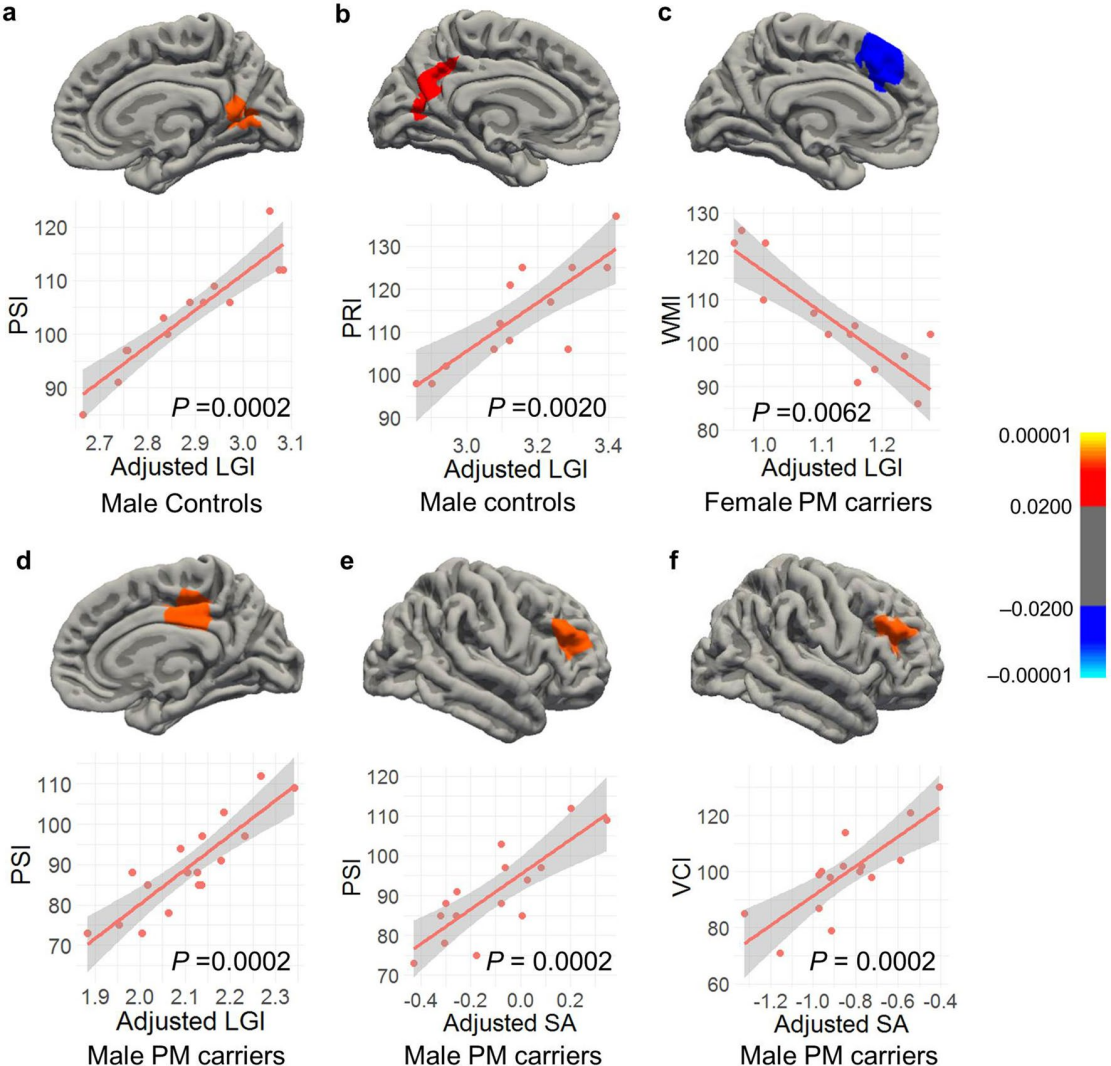
Cortical regions showing significant correlations with cognitive performance. (**a**) The positive correlation between LGI and PSI in a cluster peaked at the right precuneus in male controls. (**b**) The positive correlation between LGI and PRI of a cluster peaked at the left precuneus in male controls. (**c**) The negative correlation between LGI and WMI in a cluster peaked at the left caudal anterior cingulate cortex in female PM carriers. (**d**) The positive correlation between LGI and PSI in a cluster peaked at right posterior cingulate cortex in male PM carriers. (**e**) The positive correlation between SA and PSI in a cluster peaked at the right rostral middle frontal cortex in 15 male PM carriers all scanned using an 8-channel headcoil. (**f**) The positive correlation between SA and VCI in a cluster peaked at the right rostral middle frontal cortex in 15 male PM carriers all scanned using an 8-channel headcoil. LGIs and SA adjusted for age and total cranial volume are shown. All clusters are formed using cluster-forming threshold of *p* = 0.010. Cluster-wise *p* values are shown in the graph.

Cognitive assessment was performed using the Wechsler Intelligence Scale for Children, Fourth Edition (WISC-IV)^37^, which contains four subtests utilized in this study: namely, verbal comprehension index (VCI), perceptual reasoning index (PRI), working memory index (WMI), and processing speed index (PSI). Full scale IQ (FSIQ) of below 70 is traditionally used as a cutoff for intellectual disability. Only one PM boy had FSIQ of 51who was excluded from the analysis. The correlation of LGI, CT, and SA with WISC-IV scores was examined for the four groups separately using the whole-brain vertex-wise analysis. The WMI score (59) of a PM girl showing an outlier effect was also excluded. Setting both CFT and cluster-wise *p* value cutoffs at 0.010, significant LGI clusters peaked at the left and right precuneus showing positive correlations with PSI (cluster-wise *p* = 0.0002) and PRI (cluster-wise *p* = 0.0020), respectively, in male controls (Table 4, Fig. 3a,b). In addition, a cluster peaked at the right posterior cingulate cortex demonstrating significant positive correlations with PSI (cluster-wise *p* = 0.002) in PM boys (Fig. 3d) while a cluster peaked at the left caudal anterior cingulate showing a negative correlation with WMI (cluster-wise *p* = 0.0062) in PM girls (Fig. 3c). The only SA cluster that passed the thresholds peaked at the right rostral middle frontal cortex, showing a positive correlation with PSI in PM boys (cluster-wise *p* = 0.0080) while no significant CT clusters were detected in any of the four groups. After excluding the 4 scans acquired using a 32-channel head coil, only the two clusters of PM boys remained significant. The other three clusters were significant only at CFT of 0.05. After removing two 32-channel scans from the PM male group, a cluster centered at rostral middle frontal cortex passed the thresholds, revealing significant correlation between SA and VCI (Fig. 3f, Table 4). This cluster also passed the thresholds after adjusting for the Euler number while the PSI-SA cluster only approached significance (*p* = 0.029). All significant clusters also passed CFT of 0.005, showing cluster-wise *p* = 0.0002–0.0130 except the LGI-PRI cluster in male controls (Table 4).

**Table 4:** Correlation between cortical measurements and cognitive performance in controls and pm carriers.

Finding	Group	Brain region	Size(mm^2^)	Talairach Coordinates (x, y, z)	Cluster-Wise *P*
**Clusters survived CFT of p = 0.010 (Euler number not used as a covariate)**					
LGI and PRI (+)	Male NC	Left precuneus, extending to cuneus, pericalcarine, and lingual cortices	1349.59	− 13.3, − 50.1, 40.2	0.0020
LGI and PSI (+)	Male NC	Right precuneus, extending to isthmus cingulate, pericalcarine, and lingual cortices	1362.11	5.6, − 55.6, 16.1	0.0002
LGI and PSI (+)	Male PM carriers	Right posterior cingulate, extending to paracentral and isthmus cingulate cortices	1383.06	14.6, − 25.7, 36.6	0.0002
LGI and WMI (−)	Female PM carriers	Left caudal anterior cingulate, extending to superior frontal cortex	1034.63	− 8.2, 25.5, 25.1	0.0062
SA and PSI (+)	Male PM carriers*	Right rostral middle frontal cortex, extending to pars triangularis	1503.82	35.1, 42.4, 23.5	0.0002
SA and VCI (+)	Male PM carriers*	Right pars opercularis, extending to rostral middle frontal and pars triangularis cortices	1493.77	43.5, 25.9, 19.8	0.0002
**Clusters survived CFT of p = 0.005 (Euler number not used as a covariate)**					
LGI and PSI (+)	Male NC	Right lingual	668.2	25.4, − 59.8, 0.4	0.0122
LGI and PSI (+)	Male PM carriers	Right posterior cingulate, extending to paracentral and isthmus cingulate cortices	1191.61	14.6, − 25.7, 36.6	0.0002
LGI and WMI (−)	Female PM carriers	Left caudal anterior cingulate, extending to superior frontal cortex	687.47	− 8.2, 25.5, 25.1	0.0130
SA and PSI (+)	Male PM carriers*	Right rostral middle frontal cortex, extending to pars triangularis	729.03	35.1, 42.4, 23.5	0.0002
SA and VCI (+)	Male PM carriers*	Right rostral middle frontal cortex, extending to pars opercularis and pars triangularis	760.92	43.5, 25.9, 19.8	0.0070
**Clusters survived CFT of p = 0.010 (Euler number used as a covariate)**					
SA and VCI (+)	Male PM carriers*	Right rostral middle frontal cortex, extending to pars opercularis and pars triangularis	1262.02	43.5, 25.9, 19.8	0.0002

(+), Positive correlation; (−), negative correlatiion. *included 15 PM boys scanned with an 8-channel headcoil only. 10 mm smooth kernels for CT and SA were used.

## Discussion

The current study compared cortical morphology among 8–12 year old children with the *FMR1* normal and premutation alleles, and examined the correlations with *FMR1* molecular measures and IQ subtest scores. To the best of our knowledge, this is the first neuroimaging study on children with the PM, which aimed to understand the neurobiological basis underlying cognitive development and psychiatric conditions that might occur in a subset of this population. We revealed a group-by-age interaction of LGI in the supramarginal and inferior parietal cortices (LGI1), a group-by-sex-by-age interaction of LGI in the right inferior temporal and fusiform cortices (LGI2), a group effect (PM vs. NC) of CT in the lateral and medial orbitofrontal and frontal pole cortices (CT1), and a group-by-sex-by-age interaction of CT in the right lateral occipital cortex (CT2) (Fig. 1, Table 2). Critically, the average LGI of the LGI2 cluster correlated significantly with CGG repeat length in both boys and girls with the PM, indicating the influence of the expanded CGG repeat length on the LGI change. We also applied a novel individualized method to identify individuals with aberrant gyrification, which detected a significantly higher percentage of brain regions with aberrant gyrification in the PM carriers compared with that in the controls (*t* = − 7.12, df = 56.7, *p* < 0.0001; Table 3). PM carriers with aberrant gyrification (78.8%) included 7 boys and 2 girls with hypergyrification in 1–6 cortical regions, 9 boys and 5 girls with hypogyrification in 1–17 regions, and 3 girls with both hyper- and hypo-gyrification in 3–5 regions (Fig. 2). Controls with aberrant gyrification (14.3%) included one girl with hypergyrification and two boys and one girl with hypogyrification in only one region per person. The LGI quantification also demonstrated functional significance by showing positive correlations with PRI and PSI in male controls and PM carriers, and a negative correlation with WMI in female PM carriers in the precuneus and cingulate regions (Fig. 3, Table 4).

The *FMR1* gene is likely involved in cortical expansion and folding, the key processes in brain development that are evolutionally important and contribute to human intelligence^38^. Many early developmental processes can influence the tangential and radial growth of the cortical plate critical for gyrification, including the abundancies of progenitor cells, neurogenesis, gliogenesis, and basal radial glial cells as well as cell migration, dendritic arborization, formation of cortical connections, and modular progenitor cell density and gene expression differentiated according to regions destined to be gyri and sulci^39–41^. Mutation in genes involved in stem cell proliferation and differentiation and neuronal migration such as microtubule structural proteins (tubulin) or microtubule-associated proteins (MAPs) are associated with malformations of cortical development including lissencephaly, polymicrogyria, and periventricular nodular heterotopia^42–44^. FMRP expression starts from neurogenesis during early embryonic development in humans and is involved in neural stem cell differentiation, neuronal migration, and establishing balanced excitatory and inhibitory neuronal networks^45–47^. Loss of FMRP expression in fragile X syndrome (FXS) can lead to periventricular nodular heterotopia, the presence of nodules of gray matter along the lateral ventricles due to migration failure in some of the neurons during embryonic development^44^. Additional evidence supporting a potential link between *FMR1* gene and aberrant gyrification include the FMRP regulation of microtubule network formation and transport of mitochondria in flies^48^, disoriented neuronal migration in embryonic knock-in mouse models of the *FMR1* premutation^26^, and dysregulated global transcriptome in the second trimester of human PM fetuses using cell-free fetal RNA obtained from amniotic fluid supernatant^49^.

In this study, we provided corroborative findings, which demonstrated a high percentage (78.8%) of aberrant gyrification in PM children, characterized by variable presentation in the direction (i.e., hyper- and hypo-gyrification) and extent of affected cortical regions (1–17 regions/person) (Fig. 2b,c). Cortical regions at all levels of cortical hierarch were involved although the fusiform, inferior temporal, and medial temporal regions were more affected in PM girls than PM boys (Fig. 2a). In addition, PM boys with aberrant gyrification involving the primary sensorimotor cortices showed significantly higher proportions of aberrant LGI regions than those with aberrant LGI regions involving the associative cortices only (range 0.029–0.25 versus 0.015–0.029, *p* = 0.009). Since primary fissures formed early during cortical development may be under the highest genetic influence, while secondary and tertiary fissures formed later in the developmental processes^50–52^ are more influenced by environment and cortical plasticity^52^, both genes and environmental factors may contribute to aberrant gyrification observed across all cortical regions in the PM carriers. However, the molecular/cellular mechanisms of how PM can lead to both hyper- and hypo-gyrification are beyond the scope of current investigation and remain to be explored.

The high percentages of anxiety (80%), ADHD (50%), autistic symptoms (47.4% of the boys, 33.3% of the girls), and ID/borderline IQ (FSIQ < 80: 16.7% of the boys, 7.1% of the girls) in the PM group (Supplementary Table S1) was comparable to previous reports. However, these rates were not preferentially increased in probands in this cohort (Supplementary Table S2). In a study of 27 PM males (ages 4–22 years, 14 probands, 13 non-brobands), 93% of probands and 38% of non-probands met the criteria for ADHD while 79% of probands and 8% of non-probands had autistic symptoms^9^. In another study of 35 PM carriers (27 males, ages 5–23 years, 20 probands, 15 nonprobands), 70.6% were diagnosed with at least one anxiety disorder, and 33.3% males and 25% females had FSIQ < 80^8^. Of the 3 boys and 1 girl with FSIQ < 80 in the current study, CGG repeat length was 147 (the larger allele for the girl), 152, 157, and 182, respectively, indicating the potential influence of low FMRP expression on IQ. The numbers of regions with aberrant gyrification were 3, 4, 6, and 17 regions, respectively. Gyrification has been studied in other developmental disorders including autism, anxiety, and ADHD, which showed inconsistent results regarding the direction of gyrification change and affected cortical regions. Hypergyrification in the temporal, parietal, and frontal regions has been reported in young adults with generalized anxiety disorder^53^ while hypogyrification in the similar frontoparietal to temporal regions has been reported in another study of young adults with panic disorder^54^. Hypogyrification in the frontal, temporal, and parietal regions has been reported in cross-sectional studies of children with ADHD^55,56^, but no group difference or age-related change were observed in a large longitudinal study of 234 children with ADHD compared with 231 typically developing children^57^. Both hyper- and hypo-gyrification in different cortical regions have been reported in children with autism quantified using Freesurfer^58,59^. These inconsistencies may indicate individual variability in gyrification within the patient populations and question the adequacy of group-specific analysis in identifying aberrant gyrification. The levels of CFT and cluster-selection threshold can also influence the results. In the current study, we applied multiple cutoff values considering different levels of potential false positives and false negatives (Tables 2 and 4).

In addition, LGI measures the degree of cortex buried within sulcal folds, which is influenced by sulcal depth and brain size in addition to sulcal frequency^33,60^. Consequently, unlike folding pattern that is mostly apparent at birth and remains stable throughout the adult life^31,61^, LGI declines with age in cross-sectional studies after adjusting for brain size, which is mainly attributable to cortical flattening starting from adolescence^62–64^. Therefore, findings of LGI change can reflect changes in sulcal depth in addition to folding degree. Methods of direct comparisons of folding degree such as sulcal pit and local spectral analysis have been proposed but currently, the methods are not publicly available^64–66^. Other methodology issues that we have encountered included insufficient sensitivity of Freesurfer in detecting tiny folds preferentially presented in the cingulate and pericalcarine cortices as well as more errors in the reconstructed white matter surface in brains with aberrant gyrification that required more manual intervention than brains with normal folding patterns. Topological complexity is reflected in the Euler number^34,35^, which makes it unsuitable to serve as a covariate for movement artifacts in LGI analyses. However, we have included the Euler number in the analyses of CT and SA, which has influenced most of the results. Since LGI, CT, and SA are highly correlated with each other revealed in our results and previous studies^67^, it remains elusive currently how to untangle the complex relationships between the Euler number, movement artifacts, and topological complexity and their effects on cortical measures. In addition, it is difficult to distinguish whether the MRI changes observed in this PM sample reflecting the PM status or neuropsychiatric conditions without a second non-carrier control group with a similar rate of anxiety or ADHD. The PM sample along with the findings in the current study likely represents a subgroup of PM children who have elevated risks for neuropsychiatric conditions. More studies on unbiased sample selections from the PM population are needed to establish a clear picture of clinical involvement and associated brain alterations.

In spite of these limitations, significant clusters centered in the supramarginal, lateral occipital, and orbitofrontal cortices revealed in the group analyses of LGI and CT (Fig. 1, Table 2) were consistent with findings from PM literatures. Deficient visual motion processing in infant and adult female PM carriers have been reported^17^ as well as reduced functional activities in the temporoparietal junction (containing the supramarginal region) during temporal processing^68,69^ and in the occipital region during emotional processing in adult PM carriers^70^. In addition, the orbitofrontal cortex along with the cingulate and prefrontal cortices showing significant correlation with WISC-IV subtest scores in PM carriers (Fig. 3) are part of the neural circuits implicated in ADHD^71,72^. These regions are also important for executive control function and response inhibition documented to show subtle impairment in adult PM carriers^19,73^. Further studies are needed to investigate whether gyrification quantification can serve as a useful biomarker of fetal/infant neurodevelopment associated with cognitive development and susceptibility for neurodevelopmental conditions experienced by some PM carriers.

## Methods and materials

### Participants

Both PM carriers and controls were recruited between 2008 and 2015 from families affected by FXS or were probands who were themselves seen in our clinic. Controls were also recruited from the local community through advertisements. Informed written consent was obtained from the caregiver of each participant. All aspects of the study were approved by the Institutional Review Board of the University of California, Davis and all methods were performed in accordance with their guidelines and regulations.

Participants with PM went through detailed medical examinations, and a history of developmental and medical problems was gathered for each. PM carrier status was confirmed by *FMR1* DNA testing. CGG repeat size and methylation status were determined from genomic DNA isolated from peripheral blood lymphocytes using a combination of PCR and Southern blot analysis, as previously described^74–76^. Methylation status including percentage of methylation and for female PM carriers, the AR were measured by densitometry analysis^76^. Total RNA was isolated from 2.5 ml peripheral blood and *FMR1* mRNA expression levels were measured by quantitative Real Time PCR using a 7900 Sequence detector (Applied Biosystems) as previously described^27^. All PM carriers included in this study carried only one single PM allele and did not show methylation on the *FMR1* gene.

### MRI acquisition and analysis

A Siemens Trio 3T MRI scanner (Siemens Medical Solutions, Erlangen, Germany) was used to collect T1-weighted magnetization-prepared rapid gradient-echo (MPRAGE) scans covering the whole brain. Across 8 years of image acquisition, two different acquisition protocols were utilized. See Table 5 for details. We used visual inspection as well as two quantitative methods to assess image quality. First, we obtained the Euler number, which was calculated based on total number of defect holes (g) on the reconstructed cortical surfaces prior to topological fixing provided by Freesurfer as 2–2g. The Euler number has been reported to correlate with manual identification of “unusable” scans and show complex relationships with cortical thickness^35^. However, the Euler number, reflecting topological complexity of reconstructed cortical surface, confounded with LGI. To compliment, we generated nine image QC metrics using the LONI Quality Control System^36^, including MSI, SNR, SVNR, CNR, CVNR, TCTV, FWHM, CoM, and COV. The LONI QC metrics have been validated against expert visual inspection for multi-site and multi-scanner structural MRI scans^36^. Two additional T1 scans acquired from a 7-year-old control male and a 13-year-old PM male not included in the study because of age and excessive motion were utilized as test scans.

**Table 5:** Acquisition protocols for MPRAGE.

Head coil	*N*	# of sagittal slices	Field of view (mm^2^)	Repetition time (ms)	Echo time (ms)	Inversion time (ms)	Flip angle (°)	Image resolution (mm^3^)	Acquisition time (min)
8 channel	57	208	243	2500	4.33	1100	7	0.475	5:12
32 channel	5	192	256	2170	4.86	1100	7	1.000	8:06

For image preprocessing, the scans with the 0.475 mm^3^ image resolution were down-sampled to 1 mm^3^ using the mri_convert command from the Freesurfer software package^34,77^. All scans were then anterior–posterior commissure aligned automatically using the acpcdetect program from the Automated Registration Toolbox (www.nitrc.org/projects/art) followed by MRI bias field correction using N4^78^. LGI quantifying cortical folding complexity at each vertex of reconstructed cerebral cortex was obtained using Freesurfer version 6.0 (https://surfer.nmr.mgh.harvard.edu/)^33,34,77,79–84^. Standard procedures for cortical reconstruction were carried out automatically including motion correction, brain extraction using a hybrid watershed/surface deformation, Talairach transformation, subcortical segmentation, intensity normalization, and tessellation of the gray and white matter boundary using a deformable surface algorithm to place the boundary at the location of greatest shift in intensity. Then deformable procedures were performed for surface inflation, registration to a spherical atlas based on individual cortical folding patterns, cortical parcellation, and generation of maps of surface-based measurement which included the thickness and surface area. After the Freesurfer process was completed, the gray and white matter boundaries were checked for accuracy and appropriate files were edited according to Freesurfer’s instruction followed by automatic regeneration of the gray and whiter matter surfaces. The last two steps were reiterated until all major errors were corrected. Finally, individual surface data was registered to Freesurfer template using Freesurfer’s spherical registration.

To compute LGI^33^, an outer smoothed surface tightly wrapping the pial surface was created and the gaps between gyri on the outer surface of the brain were closed. Then tessellation of the smoothed outer surface was performed, and the LGI at a given vertex was computed as the ratio between a spherical region on the outer surface centered at the vertex and the corresponding region on the pial surface, using the default diameter, 25 mm. Regional LGI data were also available for 68 cortical regions in the left and right hemispheres using the Desikan-Killiany atlas^79,83^.

### Statistical analysis

Whole-brain vertex-wise comparisons of LGI, CT, and SA were performed to detect the effects of carrier-status, carrier-status-by-age, carrier-status-by-sex, and carrier-status-by-age-by-sex by using general linear models implemented in the mri_glmfit function of Freesurfer. As cranial size affects LGI, CT, and SA likewise^60^, Freesurfer-generated TCV was included as a covariate in all statistical models. Monte Carlo simulation was performed to correct multiple comparisons^85^. First, LGI was smoothed using FWHM Gaussian kernels of 5 mm consistent with previous studies of LGI^86^. Second, to balance the need for reducing both false positive rate and false negative rate, we examined CT and SA smoothed using 10 mm and 15 mm kernels given 10 mm as the most commonly used kernel and > 10 mm as the recommendation to reduce false positive rate^87^. Third, to reduce the data to sets of contiguous voxels with *p* values above a threshold^85^, three levels of CFT (0.05, 0.01, and 0.005) were tested and reported. The *p* value for cluster-wise correction for multiple comparisons was set to 0.05. The correlation between LGI and WISC-IV scores was examined similarly for each group excluding those with ID (FSIQ < 70, *N* = 1, a PM boy). Both the CFT and cluster-wise *p* value were set to 0.01. CFT of 0.005 was also tested for comparisons. Plots were inspected to identify potential outlier effect.

Individual participants with brain regions containing aberrant gyrification were identified using *Z*-scores relative to controls accounting for age, sex, and TCV. Individuals with Z-scores above 2.58 and below − 2.58 were considered as having hyper- and hypo-gyrification, respectively. Percentage of regions with hyper- and/or hypo-gyrification were compared between PM carriers and controls using *t*-tests. Correlations between quantitative measures were assessed using Pearson’s correlation coefficients. The analyses were conducted using the open-source statistical package R (https://www.r-project.org/).

## Supplementary information

Supplementary Information.
