# Supplementary material for: Cortical gyrification and its relationships with molecular measures and cognition in children with the FMR1 premutation
Source: Sci Rep. 2020 Sep 29;10:16059. doi: 10.1038/s41598-020-73040-0 (PMC7525519; doi:10.1038/s41598-020-73040-0)
Supplement: Supplementary file 1 — Supplementary Information. [file 41598_2020_73040_MOESM1_ESM.docx]

**Supplementary Information**

J.Y. Wang, M. Danial, C. Soleymanzadeh, B. Kim, Y. Xia, K. Kim, F. Tassone, R.J. Hagerman, and S.M. Rivera. “Cortical gyrification and its relationships with molecular measures and cognition in children with the *FMR1* premutation”

| **ID** | **Sex** | **Age** | **Proband** | **CGG1** | **CGG2** | **AR** | **mRNA** | **FSIQ** | **VCI** | **PRI** | **WMI** | **PSI** | **Autism** | **Anxiety** | **ADHD** | **Hyper (N)** | **Hypo (N)** |
| --- | --- | --- | --- | --- | --- | --- | --- | --- | --- | --- | --- | --- | --- | --- | --- | --- | --- |
| F1 | f | 7.85 | No | 30 | 76 | 0.78 | NA | 111 | 119 | 121 | 94 | 91 | No | No | No | 0 | 0 |
| F2 | f | 8.51 | No | 32 | 110 | 0.2 | 3.238 | 111 | 108 | 110 | 110 | 103 | Yes | Yes | Yes | 0 | 0 |
| F3 | f | 8.74 | No | 37 | 61 | 0.52 | 1.603 | 101 | 100 | 110 | 102 | 83 | No | Yes | No | 1 | 0 |
| F4 | f | 9.28 | No | 52 | 110 | 0.5 | 5.19 | 130 | 119 | 133 | 126 | 112 | Yes | No | No | 2 | 3 |
| F5 | f | 9.67 | No | 31 | 63 | 0.5 | 1.98 | 102 | 96 | 108 | 104 | 94 | No | Yes | No | 2 | 0 |
| F6 | f | 10.29 | No | 20 | 65 | 0.44 | 1.849 | 107 | 110 | 112 | 97 | 94 | No | Yes | Yes | 0 | 0 |
| F7 | f | 10.54 | Yes | 30 | 77 | 0.63 | 1.493 | 134 | 124 | 133 | 123 | 126 | No | Yes | No | 0 | 3 |
| F8 | f | 10.7 | No | 33 | 69 | 0.52 | 1.881 | 109 | 119 | 100 | 102 | 103 | No | Yes | Yes | 0 | 1 |
| F9 | f | 11.67 | No | 29 | 89 | 0.28 | 2.287 | 106 | 104 | 112 | 86 | 112 | NA | NA | NA | 0 | 0 |
| F10 | f | 12.09 | No | 31 | 140 | 0.52 | 2.184 | 124 | 132 | 125 | 102 | 109 | No | No | No | 2 | 2 |
| F11 | f | 12.23 | No | 30 | 147 | 0.27 | 2.902 | 76 | 85 | 98 | 59 | 75 | Yes | Yes | No | 1 | 2 |
| F12 | f | 12.41 | No | 21 | 79 | 0.56 | 1.92 | 91 | 110 | 94 | 91 | 70 | NA | NA | NA | 0 | 3 |
| F13 | f | 12.54 | No | 31 | 110 | 0.48 | 2.08 | 124 | 140 | 121 | 107 | 97 | No | NA | NA | 0 | 17 |
| F14 | f | 12.95 | Yes | 23 | 142 | 0.48 | 3.26 | 120 | 116 | 110 | 123 | 115 | Yes | No | No | 0 | 4 |
| M1 | m | 8.5 | Yes | 147 | - | - | 2.62 | 84 | 79 | 108 | 68 | 88 | Yes | Yes | Yes | 0 | 1 |
| M2 | m | 8.54 | No | 124 | - | - | 3.69 | 93 | 99 | 102 | 86 | 88 | No | Yes | Yes | 0 | 0 |
| M3 | m | 8.55 | Yes | 157 | - | - | 5.05 | 73 | 71 | 90 | 68 | 85 | Yes | Yes | Yes | 6 | 0 |
| M4 | m | 8.84 | Yes | 77 | - | - | 2.174 | 108 | 114 | 108 | 99 | 97 | No | Yes | Yes | 1 | 0 |
| M5 | m | 9.73 | Yes | 120 | - | - | 4.001 | 89 | 102 | 94 | 91 | 75 | Yes | Yes | No | 0 | 0 |
| M6 | m | 9.78 | Yes | 73 | - | - | 2.54 | 105 | 100 | 106 | 102 | 103 | No | Yes | No | 0 | 0 |
| M7 | m | 10.24 | No | 102 | - | - | 2.666 | 119 | 130 | 108 | 104 | 109 | No | Yes | Yes | 1 | 0 |
| M8 | m | 10.5 | Yes | 74 | - | - | 1.97 | 82 | 85 | 94 | 91 | 73 | Yes | No | Yes | 0 | 10 |
| M9 | m | 10.75 | No | 67 | - | - | 2.16 | 102 | 99 | 104 | 113 | 88 | Yes | Yes | Yes | 0 | 2 |
| M10 | m | 10.95 | No | 182 | - | - | 3.61 | 70 | 71 | 88 | 65 | 73 | Yes | Yes | Yes | 0 | 17 |
| M11 | m | 11.08 | No | 71 | - | - | 1.99 | 92 | 98 | 96 | 88 | 94 | No | Yes | Yes | 1 | 0 |
| M12 | m | 11.16 | Yes | 90 | - | - | 2.19 | 109 | 121 | 108 | 97 | 97 | No | Yes | No | 0 | 5 |
| M13 | m | 11.33 | No | 110 | - | - | 3.701 | NA | NA | NA | NA | NA | No | Yes | Yes | 0 | 2 |
| M14 | m | 11.66 | Yes | 55 | - | - | 1.469 | 84 | 87 | 104 | 77 | 78 | Yes | Yes | Yes | 0 | 1 |
| M15 | m | 11.71 | No | 70 | - | - | 1.913 | 100 | 100 | 104 | 99 | 91 | No | Yes | No | 3 | 0 |
| M16 | m | 11.84 | No | 102 | - | - | 3.497 | 100 | 102 | 102 | 104 | 85 | No | Yes | Yes | 0 | 3 |
| M17 | m | 11.86 | Yes | 152 | - | - | 2.952 | 51 | 65 | 53 | 59 | 62 | Yes | No | No | 0 | 4 |
| M18 | m | 12.01 | Yes | 65 | - | - | 1.923 | 87 | 98 | 88 | 86 | 85 | Yes | Yes | No | 2 | 0 |
| M19 | m | 12.36 | No | 65 | - | - | 2.45 | 113 | 104 | 112 | 110 | 112 | No | Yes | No | 4 | 0 |

**Supplementary Table S1.** Detailed information of the 33 premutation carriers.

| ***N* (Rate)** | **Anxiety** | **ADHD** | **Anxiety & ADHD** | **FSIQ < 80** |
| --- | --- | --- | --- | --- |
| Nonprobands | 16 (0.84) | 11 (0.58) | 11 (0.58) | 1 (0.11) |
| Probands | 8 (0.73) | 4 (0.36) | 3 (0.27) | 2 (0.18) |

**Supplementary Table S2.** Number and rate of anxiety, ADHD, and FSIQ < 80 among probands and nonprobands.


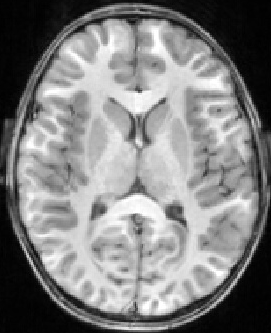


8-year-old PM boy

Euler -334


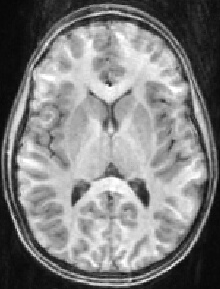

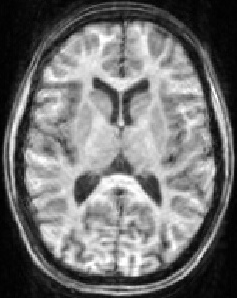


**a**

**b**

**c**

7-year-old NC boy

Euler -330

13-year-old PM boy

Euler -468

Supplementary Fig. S1 Euler number and movement artifacts


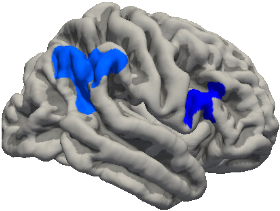

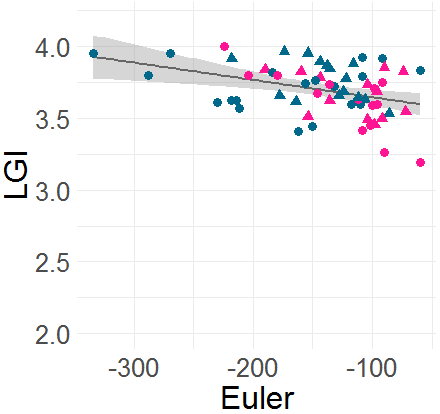

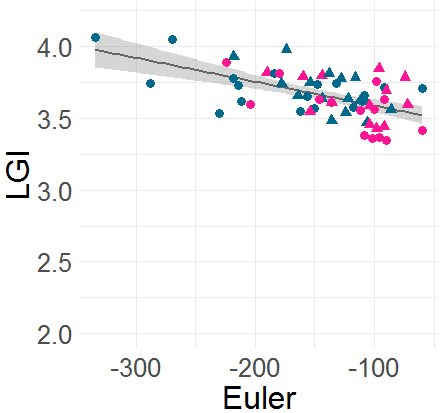

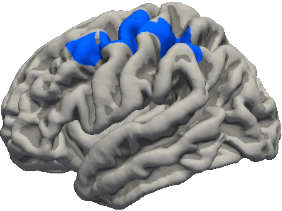


*p* =0.0002

LGI of left precentral

**a**

–0.00001

0.00001

0.0200

–0.0200


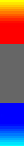


*p* =0.0002

**b**

LGI of right IP & RMF


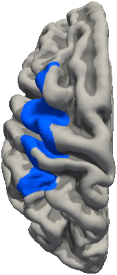

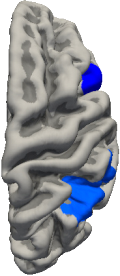

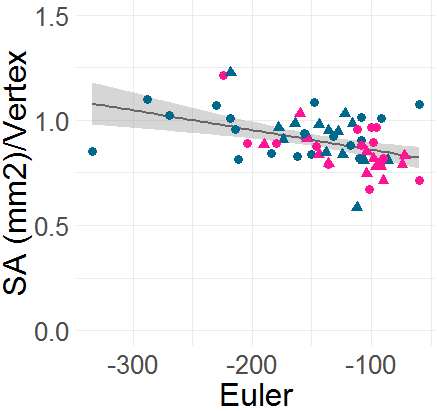

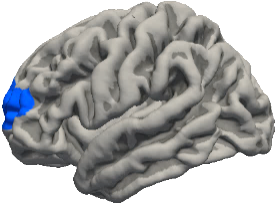


**c**

*p* =0.0002

SA of left SF


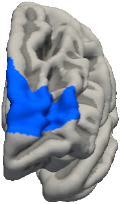


F

NC

M

PM


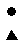

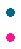


Supplementary Fig. S2 Effect of the Euler number on cortical measures. (a) A cluster peaked at the left precentral cortex showing the effect of the Euler number on LGI. (b) Two clusters peaked at the right inferior parietal (IP) and rostral middle frontal (RMF) cortices, respectively, show the effect of the Euler number on LGI. The graph on the right shows the relationship between LGI of the IP cluster and the Euler number. (c) A cluster peaked at the left superior frontal (SF) cortex shows the effect of the Euler number on SA.

Supplementary Fig. S3 The correlation between the average LGI of cluster LGI2 and CGG repeat length in male and female PM carriers. (a) The correlation between CGG repeat length and LGI in male PM carriers after adjusting for age and TCV. (b) The correlation between CGG repeat length and LGI in female PM carriers after adjusting for age, TCV, and activation ratio. (c) The correlation between activation ratio and LGI in female PM carriers after adjusting for age, TCV, and CGG repeat length.


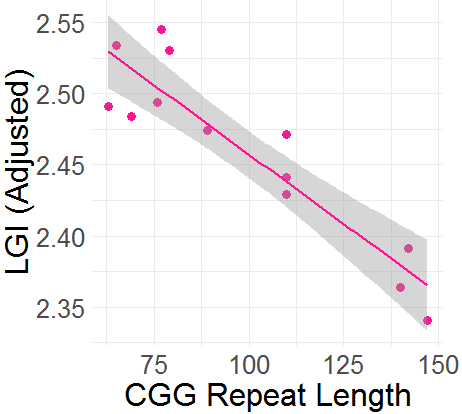

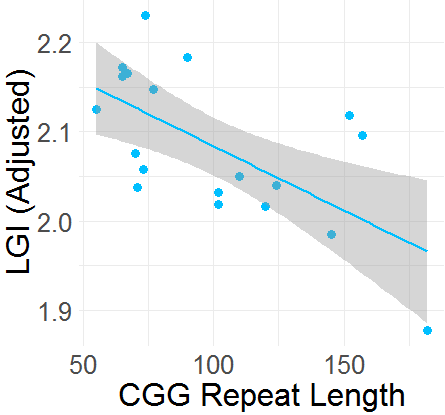


**a**

**b**

*P* =0.010

*P* =0.001


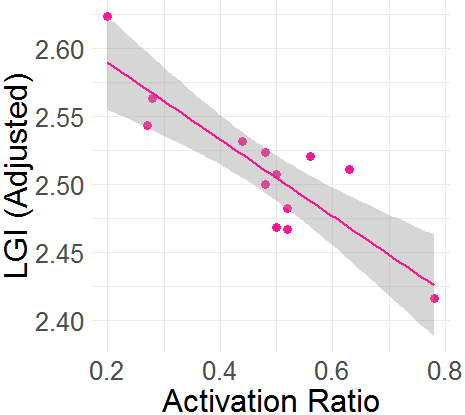


**c**

*P* =0.002
